# Understanding the Damage Mechanisms of Basalt/Carbon Fiber Hybrid Composites Under Quasi-Static and Dynamic Loadings

**DOI:** 10.3390/polym17070866

**Published:** 2025-03-24

**Authors:** Mehmet İskender Özsoy, Sinan Fidan, Mustafa Özgür Bora, Satılmış Ürgün

**Affiliations:** 1 Department of Mechanical Engineering, Faculty of Engineering, Sakarya University, Sakarya 54050, Türkiye; 2 Department of Airframe & Powerplant Maintenance, Faculty of Aeronautics and Astronautics, Kocaeli University, Kocaeli 41001, Türkiye; sfidan@kocaeli.edu.tr (S.F.); ozgur.bora@kocaeli.edu.tr (M.Ö.B.); 3 Department of Aviation Electrics and Electronics, Faculty of Aeronautics and Astronautics, Kocaeli University, Kocaeli 41001, Türkiye; urgun@kocaeli.edu.tr

**Keywords:** hybrid composites, carbon fiber, basalt fiber, flexural properties, impact resistance

## Abstract

This work investigates the hybrid fiber sequence effect on the flexural and impact properties of basalt/carbon epoxy composites. In the present study, six configurations of composite laminates were fabricated by vacuum-assisted resin transfer method and tested in three-point bending and Charpy impact tests. The results show that hybrid composites outperform pure basalt or carbon laminates. The maximum flexural strength and modulus, such as in [C_2_B_4_C_2_], were realized for the configurations with carbon fibers on the outer layers because of the rigidity of carbon. However, higher energy absorption was offered by the basalt-rich composites because of their ductility. Among the hybrids, a balanced stacking sequence like [C_4_B_4_] and [B_2_C_4_B_2_] showed an optimum between stiffness and toughness. Flexural modulus was maximum at 12.1 GPa for carbon-dominant layers, whereas impact resistance was maximum for alternating hybrid layers at 120 kJ/m^2^. SEM analysis revealed that the dominant mechanisms of failure were delamination at the fiber–matrix interface and fiber pull-out, while the stacking order was critical regarding stress distribution. Hybridization also increased cost-performance metrics by a factor of 40%, as basalt fibers reduced the cost while maintaining acceptable mechanical properties. These results prove the potential of basalt-carbon hybrid for applications requiring high strength, impact resistance, and economic efficiency.

## 1. Introduction

Fiber-reinforced polymer matrix composites (FRPMC) have low lifecycle costs; their mechanical performance is good and lightweight among the many undeniable benefits of FRPs which have been becoming the most preferred materials in structural components due to their remarkable strength, high modulus, fatigue resistance, low density, and notable chemical stability [1,2,3,4,5,6,7]. However, researchers using hybrid composites with incorporate two or more fiber types as reinforcement, either by alternating layers of distinct fibers or by intermixing inside a single layer. Hybrid design helps in the generation of materials suitable for various industrial sectors by allowing balancing between cost and functionality. Hybridization can be carried out to realize composites meeting various operating requirements, which again proves the flexibility and effectiveness of this technique in the domain of material engineering [8,9]. Hybridization may reduce several disadvantages of natural and synthetic fibers and improve environmental sustainability by using many fiber types in one matrix by enhancing the load-bearing capacity and absorption of impact energy, thus making hybrid composite an attractive alternative for applications in which high performance is needed under stress. Hybrid composites are quite exciting, as they combine the complementary qualities of different fibers that aim to attain a balance between strength and resilience with environmental friendliness. Namely, optimal mechanical qualities are hardly achieved by one fiber type, but their hybrid succeeds here [10,11,12,13,14].

Margabandu and Subramaniam [15] conducted a study on fabric stacking patterns as regards their results on flexural and impact behavior of jute/carbon hybrid epoxy composites and reported flexural strength values ranging from 78 to 142 MPa, depending on carbon fiber placement. In another study [16], impact and flexural responses of two lay-up configurations of CFRPs are investigated. Although rarely used in structural applications, the unidirectional laminates give useful information about the damage mechanisms like splitting, matrix cracking, and delamination under low-velocity impact and flexural loads. Multidirectional laminates are more complicated in their behavior due to the influence of ply orientation and coupling effects, and various failure scenarios were observed. Meliande et al. [17] investigate flexural, impact and elastic properties of non-woven curaua–aramid hybrid epoxy composites. As curaua layers progressively replaced the aramid fraction, it resulted in a 56% reduction in flexural modulus and a 33% decrease in flexural strength. In the Charpy impact tests, it was observed that as the percentage of curaua increased, the energy absorbed decreased proportionately. Chen et al. [18] conducted the interply hybridization of CFRP composite laminates to enhance the flexural performance and cost effectiveness. They noted that the introduction of basalt fibers in carbon laminates resulted in flexural modulus values between 18 and 25 GPa, significantly influenced by the hybrid ratio and stacking sequence. The carbon layers in these laminates are partially substituted with basalt and/or glass fibers in studies on the effects of hybrid ratio and stacking sequence on flexural behavior and material utilization. Najafi et al. present, in [19], the effects of impact and flexural properties of woven basalt fiber/phenolic, woven carbon fiber/phenolic and woven basalt/carbon hybrid phenolic composites. They explored woven basalt/carbon phenolic composites, where basalt fibers considerably improved impact energy absorption, reaching values around 60–90 kJ/m^2^. Basalt fiber significantly increases the impact energy absorption according to the obtained results. Boobalan et al. [20] investigated the flexural and impact properties of basalt/glass fiber-reinforced epoxy composites (flexural strength ranging between 85 and 115 MPa) enhanced with multiwalled carbon nanotubes and nano-silica. The results showed that adding 3% filler concentration resulted in the agglomeration of particles, reducing strength due to weak interfacial contact between fiber and matrix. Sun et al. [21] investigated the influence of hybrid ratios and stacking sequences on the tensile and flexural properties of carbon/basalt/epoxy hybrid composites. Subagia et al. [22] studied the effects of varying the stacking sequence when incorporating basalt fiber into carbon fiber reinforced composites and their response to flexural loading. Carbon fiber layers on the compressive side achieved higher modulus and flexural strength. Subagia and Kim [23] performed flexural testing of carbon/basalt/epoxy interply hybrid laminates, and they were able to estimate the flexural strength and modulus by taking into account the number of basalt layers. The laminates had higher flexural strength and modulus when carbon layers were on the outermost surfaces compared to when the carbon layers are on the innermost surfaces. Mithilesh and Dutt [24] present glass fiber and basalt hybrid composites. Carbon black nanoparticles were used as reinforcement, along with glass and basalt woven fibers in a vinyl ester resin matrix. The results have shown the great influence of adding carbon black to the mechanical properties of the composite and the importance of performance due to the stacking order. For a stack of G/3B/G/3B/G, the increase in flexural strength was 32.38%, while for B/3G/B/3G/B it dropped to 72.5%. Composites reinforced with basalt and 6% carbon black had the maximum impact strength. Velumayil et al. [25] investigated the neat and hybrid basalt/Kevlar-epoxy composites according to fiber volume fractions, different stacking sequences, and associated effects on modulus, hardness, tensile, flexural, and impact strengths. Subramanian et al. [26] discuss the studies related to the mechanical properties of hybrid basalt fibers with natural fiber reinforcement against pure basalt composites. Hybrid composite compression strength performed better than (up to 205 MPa) pure basalt composites. Aloe vera/basalt-reinforced polymer composite outperformed both banana/basalt-reinforced polymer composite and basalt fiber-reinforced polymer composite in terms of flexural strength among the hybrids. Lim et al. [27] discusses the stacking sequence effect of hybrid composites with carbon and basalt on flexural and fracture characteristics. Carbon layers associated with the outer skin provide improved flexural properties. Bozkurt et al. [28] studied the effects of basalt layer location on impact properties and hybridization effects of Charpy impact behavior of basalt/aramid hybrid fiber composites. Lower impact energies have provided laminates whose basalt layers were on the impacted surface and were likely caused by limited aramid layer deformation. Arpatappeh et al. [29] assessed the degradation performance of basalt and Kevlar fiber stacking configurations on Charpy impact properties of hybrid epoxy composites. Although the hybrid configuration performed better in impact behavior than neat composites, such as BKBBKB and KBKKBK configurations absorbed 26% and 12% more energy, respectively, compared with basalt-only composites and 99% and 77% more than Kevlar-only composites. Bozkurt [30] investigated the aramid fiber-reinforced polymer composites. Hybrid composites are less expensive than AFRP laminates, underscoring the advantages of hybridization for both cost-effectiveness and performance improvement. Pandian et al. [31] stated that the basalt fiber-woven laminates are marginally better than jute fiber-reinforced hybrid laminates. Hybrid composites have a major impact on impact strength, and among basalt-jute fiber combinations, the B/J/B stacking sequence exhibits the highest impact resistance. The composite with a basalt/jute alternately stacked sequence showed maximum flexural modulus and flexural strength in flexural tests. Basalt fibers, with a volcanic origin and derived from basalt rock, possess good mechanical properties like high tensile strength, good thermal and chemical stability, and high energy absorption. Used as cost-effective and eco-friendly alternatives to traditional synthetic fibers like glass or carbon, basalt fibers extensively find application in structural composite applications with a balance between ductility, strength, and resistance to impact. Their durability, along with cost and environmental advantages, makes basalt fibers extremely viable in automotive, aeronautical, and construction industries.

As seen in the literature, hybrid composites have lately been considered for cost-effectiveness, considering mechanical performance. However, very few detailed insights have been found in the literature on how different stacking sequences and fiber-type hybridization-plain weave carbon and basalt-would affect the quasi-static flexural and fracture behavior of hybrid composite configurations and their corresponding cost-performance relationships. The present work highlights these shortcomings and explores the composite combinations of carbon and basalt plain weaves in the following sequence: [C]_8_, [C_4_B_4_], [C_2_B_4_C_2_], [B]_8_, [B_4_C_4_], [B_2_C_4_B_2_]. Composite laminates, fabricated by vacuum-assisted resin transfer method, underwent flexural strength, modulus, and fracture resistance testing based on varying stacking sequences and fiber type. Further, basalt fiber content is linked to cost–performance metrics, such as cost-to-strength, -modulus, and -fracture resistance ratios. Incorporating basalt fibers into hybrid fiber-reinforced polymer matrix composites (PMCs) is advantageous because it enhances ductility, energy absorption, and impact resistance, while significantly improving cost-performance efficiency by reducing material costs without compromising mechanical integrity. Damage modes have also been investigated with high-resolution digital imaging and SEM analyses. To this extent, hybrid configurations provide a primary overview of their different impact on performance and economic efficiency.

## 2. Materials and Methods

### 2.1. Materials

**Figure 1:**
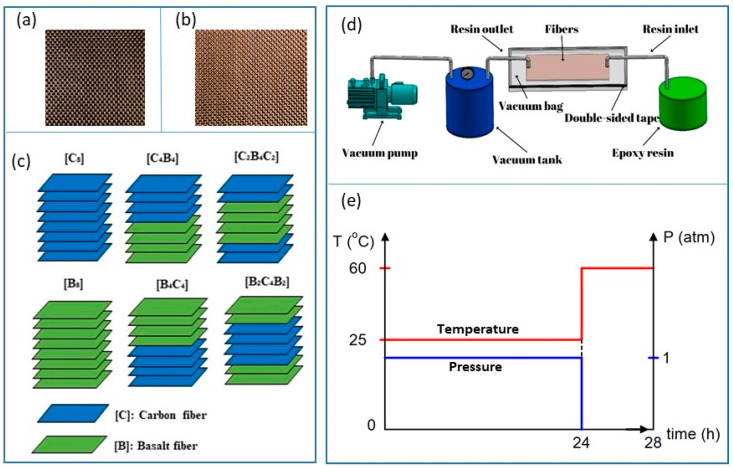
(**a**) Carbon fiber, (**b**) basalt fiber, (**c**) vacuum-assisted resin transfer process, (**d**) fiber sequences of the laminate composites, (**e**) temperature–time diagram of the manufacturing process.

Carbon and basalt plain weaves which have the areal density of 200 g/m^2^ were used in this study, as seen in Figure 1a,b. The combination of basalt and carbon fibers was selected due to their complementary properties; carbon fibers provide high stiffness and strength, while basalt fibers contribute enhanced ductility and energy absorption, leading to an optimal balance between mechanical performance and economic efficiency. Carbon and basalt plain weaves used in the study were supplied by Dost chemical corporation, İstanbul, Türkiye for the purposes of the present study. For the Sika epoxy CR80 resin and Sika CH80-2 hardener, Tekno Endüstriyel Kimyasallar San. Tic. Ltd. Şti., İstanbul, Türkiye. The resin utilized in this study is characterized by the following mechanical properties: a density of 1.01 g/mL, tensile strength of 83 MPa, and tensile modulus of 2900 MPa. It exhibits a tensile elongation at break of 5.8%, impact resistance of 29 kJ/m^2^, and Shore D hardness of 84. Additionally, the glass transition temperature of the resin was measured to be 93 °C. The carbon fiber fabric used was a 200 gr/m^2^ 3 k plain weave, specifically Tenax-E HTA 40 3 k fibers in both warp and weft directions supplied by Dost chemical corporation, İstanbul, Türkiye. This fabric has a laminate thickness of 0.327 mm, with individual fiber diameters measuring 7 µm. Its tensile strength is 3950 MPa, with an elastic modulus of 238 GPa and a strain at break of 1.7%. The thermal conductivity of these fibers is 17 W/m, and their coefficient of thermal expansion is −0.1. They exhibit a specific electrical resistivity of 1.6 × 10^−3^ Ω·cm and a density of 1.76 g/cm^3^. The basalt fiber fabric utilized was also 200 g/m^2^, characterized by a bidirectional weave structure. Its density ranges from 2.60 to 2.63 g/cm^3^, and the fiber diameter is 13 µm. With an approximate yarn number of 100 ± 2% Tex, the weave density is 10 ± 5% per cm for both warp and weft directions. The basalt fibers exhibit a tensile strength of about 3100 MPa, an elasticity modulus ranging from 88 to 92 GPa, and an elongation at break of approximately 3.5%. All the constituents in this work have been chosen in their respective domains of compatibility and well-documented credibility in composite manufacturing. Sika epoxy CR80 resin and Sika CH80-2 hardener were used as matrix material. Composites were manufactured as [C]_8_, [C_4_B_4_], [C_2_B_4_C_2_], [B]_8_, [B_4_C_4_], and [B_2_C_4_B_2_] sequences by the vacuum-assisted resin transfer method as 400 × 400 mm^2^ dimensions (Figure 1c). After the composites were cured at room conditions for 24 h, they were subjected to post-curing at 60 °C for 4 h. Then, composite plates were cut to sample sizes with a water jet machine. Figure 1d shows the fiber sequences of the laminate composites. The laminate thicknesses of the composite samples were measured as follows: 1.95 mm for C_8_, 1.5 mm for B_8_, 1.84 mm for B_2_C_6_, 1.78 mm for B_4_C_4_, 1.62 mm for B_6_C_2_ and C_2_B_6_, 1.78 mm for C_4_B_4_ and B_2_C_4_B_2_, and 1.80 mm for C_2_B_4_C_2_. The fiber volume fraction was maintained at a constant with 55% fiber and 45% resin for all composites.

In this study, six specific configurations ([C_8_], [C_4_B_4_], [C_2_B_4_C_2_], [B_8_], [B_4_C_4_], [B_2_C_4_B_2_]) were chosen to systematically investigate how different stacking sequences of basalt and carbon fibers influence the flexural and impact properties of hybrid composites. According to the article, these six configurations were selected to represent a comprehensive range of fiber arrangements, covering extremes of purely basalt or carbon fibers and various balanced hybrid configurations. This allowed the authors to investigate clearly how the positioning of fibers within the laminate (either as outer or core layers) influences stiffness, strength, and energy absorption characteristics, while also effectively highlighting the cost–performance relationships of each configuration.

**Figure 2:**
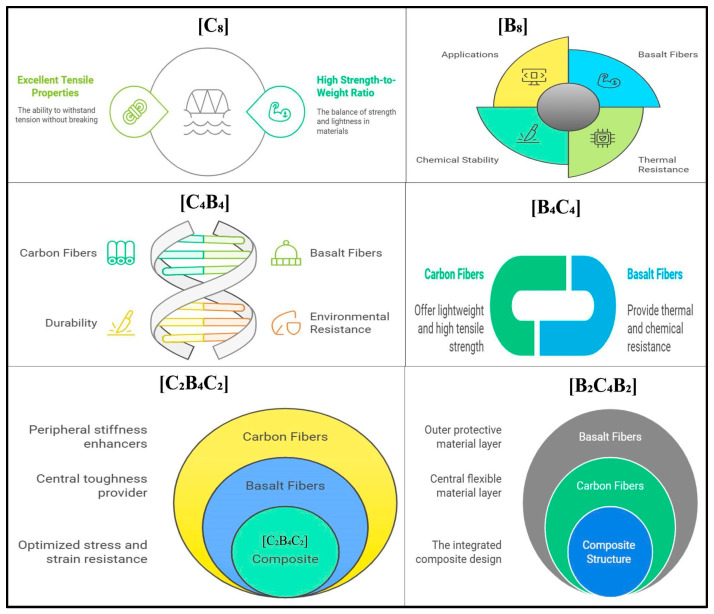
Comparison of the effects of the arrangement of basalt and carbon fiber reinforcement layers on the properties of composite materials.

Figure 2 illustrates the carbon and basalt fibers structurally and functionally incorporated into composite materials to reinforce performance characteristics. In the case of [C_8_], carbon and basalt fibers have been identified to possess high tensile strength and high strength-to-weight ratio and hence find applications in situations requiring lightweight yet strong materials. Segment [B_8_] describes the main characteristics of basalt fibers: chemical stability and thermal resistance enable a wide variety of industrial applications. The complementary nature of carbon and basalt fibers in [C_4_B_4_] is underlined, with the durability of carbon fibers combined with basalt fibers prone to environmental resistance to give strength to the composite structure. The interconnected roles of these fibers are shown in [B_4_C_4_], where carbon fibers provide tensile strength while basalt fibers contribute resistance to thermal and chemical degradation. The layered composite structure is further detailed in [C_2_B_4_C_2_], where carbon and basalt fibers are arranged to optimize peripheral stiffness and central toughness, thus enhancing the composite’s resistance to stress and strain. Finally, [B_2_C_4_B_2_] describes a layered design, with basalt fibers forming the outer protective layer, carbon fibers constituting the flexible core, and an integrated composite structure providing cohesive performance. This figure illustrates the synergy between carbon and basalt fibers, resulting in a composite that combines lightweight properties, structural toughness, and environmental resilience.

### 2.2. Mechanical Tests

**Figure 3:**
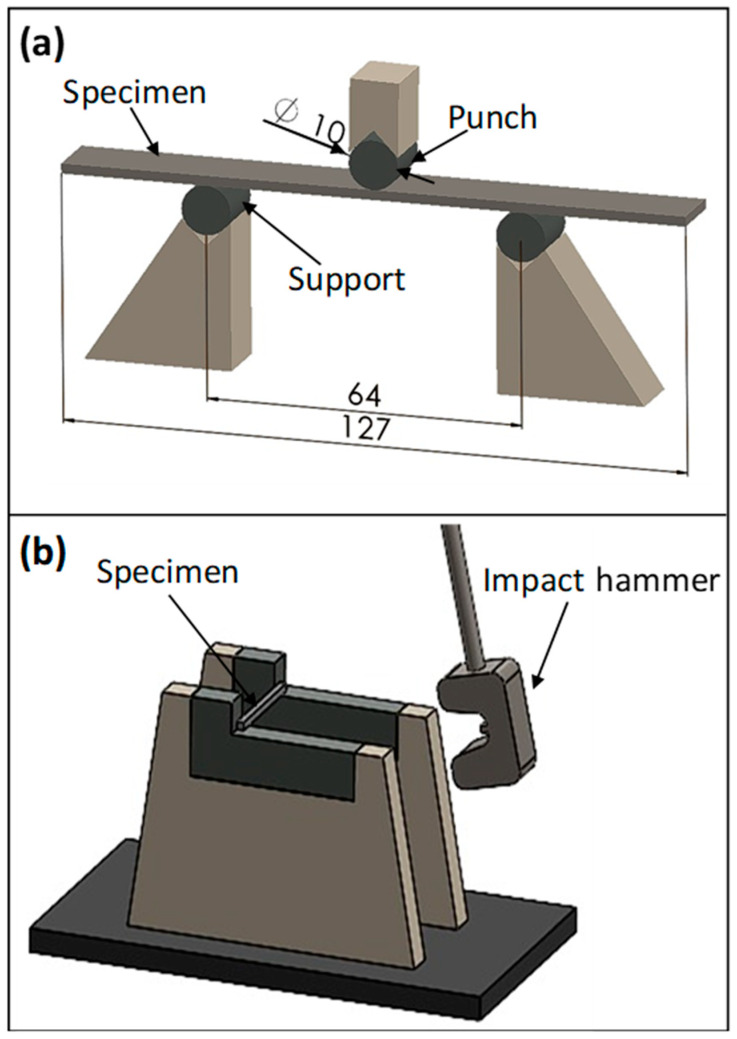
(**a**) Bending and (**b**) impact tests configurations. (all the units in Figure 3 are mm).

Mechanical tests consist of bending and Charpy impact tests. Flexural tests were carried out on a Shimadzu universal testing machine which is located in the laboratory of Sakarya University Technology Faculty in Sakarya, Türkiye. Test speeds are 2 mm/min, according to the ASTM D790-10 standard [32]. The bending test samples were 12.7 mm × 127 mm in size. The span length was taken as 64 mm. Charpy impact tests were carried out according to ISO 179-1 standard [33] with the Zwick-brand pendulum impact tester which is located in the laboratory of Marmara University Technology Faculty in İstanbul, Türkiye. The hammer capacity is 15 J. Sample dimensions are 80 mm × 10 mm. Figure 3 shows the bending and impact test configurations. Each test under bending and impact loading was repeated five times.

## 3. Results and Discussions

**Figure 4:**
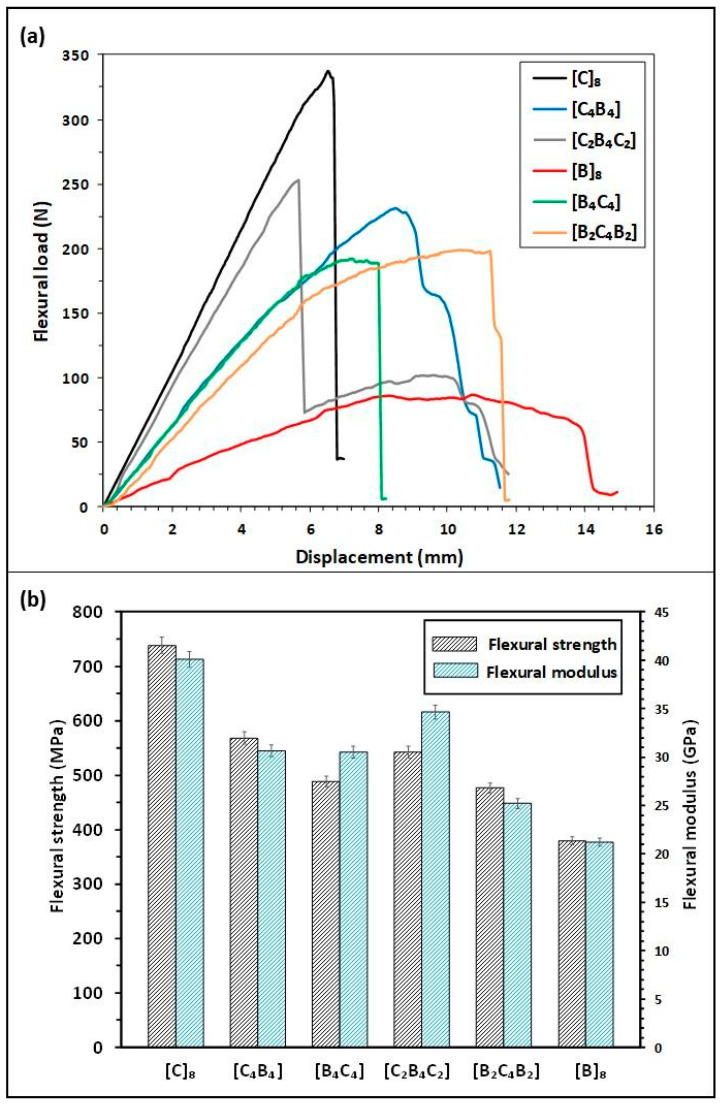
(**a**) Load–displacement curves of the composites under bending, (**b**) flexural strength and flexural modulus of the composites.

Figure 4a presents the flexural load–displacement curves of three-point bending tests on various basalt/carbon hybrid composite configurations manufactured by different ply sequences. Each of these curves shows variations in the stacking pattern, with certain differences regarding the layers of both basalt and carbon fibers. Initial stiffness and load-bearing capacity was optimum for composites which had carbon fibers on the outermost layers. This is due to the stiffness of the carbon fibers, which makes them more resistant to deformation early in the loading process. The rigid carbon fiber curve has the highest steepness. The addition of carbon fiber to the upper surfaces of the basalt fibers brings about an increase in the stiffness of the composites. Where carbon fibers are predominant or make up the outer layers, the resistance of the latter to the application of failed loads reflects their improved stiffness and strength. Because carbon fibers have low elasticity, less displacement under load is developed in these carbon-rich structures. In contrast, basalt fibers exhibit higher flexibility; hence, the stacking sequences with these fibers on top have presented a more gradual stress increase. Peak loads of these experiments show that the composites with alternating carbon–basalt layers work well because of toughness in basalt fibers, combined with the stiffness of carbon fibers, increasing energy absorption and load-carrying capability. This is because the basalt fibers are naturally tough and flexible; hence, the composite can absorb more energy before rupture compared to the carbon-rich designs. This leads to higher displacement but at a lower peak load capacity. On the other hand, hybrid structures that possess an optimum composition balance feature: an adequate amount of basalt and carbon layers. In that respect, the composite effectively attains the optimum performance related to load-carrying capability and displacement range while utilizing the high load-carrying capacity of carbon fibers but preserving the ductility and energy-absorbing qualities of basalt fibers.

The variation in flexural strength and flexural modulus of the basalt/carbon hybrid composites throughout various stacking sequences is depicted in Figure 4b. Composites with a larger carbon fiber layer ratio showed the highest modulus and flexural strength, especially when placed on the exterior surfaces. Given that this is a high rigidity realm, it would mean that carbon fibers make a significant contribution to flexural stiffness and strength. However, basalt fiber-based composites in the outer layers, due to increasing ductility with lower rigidity, reflected lower values for flexural strength and modulus. This layout allows higher strain; the stiffness becomes lower. Interestingly, the hybrid structures with a uniform arrangement of the two fibers, namely basalt and carbon, showed balanced flexural characteristics and, thus, were suitable for applications requiring both stiffness and flexibility. The highest bending performance is obtained with a hybridization ratio of 50% among hybrid composites. Composites with carbon fiber on the outer surfaces have greater load carrying capabilities. The presence of fibers with high rigidity on the outer surfaces increases the load-carrying capabilities of hybrid structures and their bending strength is greater than composites with basalt fibers on the outer surfaces. Similarly, the strain of composites with carbon fibers in the upper region is lower than others because the rigidity of these composites is higher [34]. The bending performance of sandwich type composites with glass fibers on the inside is lower than composites with glass fibers on the outside. The bending strength of sandwich type composites is higher in hybrid composites with the same number of carbon fibers. A similar situation is also seen in ref. [22].

Composite stress distribution highlights the influence of fiber type, especially in flax/epoxy layers. Although flax/epoxy plies (layers 3–5) show reduced stress due to lower stiffness—even with an equal fiber volume fraction of 30%—their lower strength restricts performance. Despite significant tensile-side failure potential, analyses indicate initial failure is most probable on the compressive side [35]. Tehrani-Dehkordi et al. [36] compare pure nylon and basalt laminates. Due to basalt’s brittle nature, the basalt laminate (M) has the highest load, steepest slope, and lowest deflection. In contrast, nylon’s high elongation results in lower load but greater deflection. Intra-ply hybrid laminates (A, B, C) exhibit intermediate loads and continued support after yielding, with load drops indicating matrix failure and delamination. Hashim et al. [37] examine the impact of ply orientation and stacking order on the flexural properties of PALF/carbon hybrid laminates. Composite stress distribution highlights the influence of fiber type, especially in flax/epoxy layers. Although flax/epoxy plies (layers 3–5) show reduced stress due to lower stiffness—even with an equal fiber volume fraction of 30%—their lower strength restricts performance. Despite significant tensile-side failure potential, analyses indicate initial failure is most probable on the compressive side [35]. Tehrani-Dehkordi et al. [36] compare pure nylon and basalt laminates. Due to basalt’s brittle nature, the basalt laminate (M) has the highest load, steepest slope, and lowest deflection. In contrast, nylon’s high elongation results in lower load but greater deflection. Intra-ply hybrid laminates (A, B, C) exhibit intermediate loads and continued support after yielding, with load drops indicating matrix failure and delamination. Hashim et al. [37] examine the impact of ply orientation and stacking order on the flexural properties of PALF/carbon hybrid laminates. Maximum flexural modulus and strength were achieved in laminates with outermost layers and [0°, 90°] orientation as outer layers of carbon can withstand loads in an optimal manner and hence enhance stiffness and strength. Stiff outer layers and perpendicular fibers give maximum flexural performance and are used in structures that need high stiffness and strength for structural integrity. Selver et al. [38] present how fiber type, volume, and sequence affect hybrid flax and jute laminate flexural strength. Outer glass fibers (GF, GJ) in laminates produced higher strengths than central glass fibers (FG, JG) due to increased bending resistance. Central glass fibers lowered strength to 50% in jute and 39% in flax hybrid. Stronger fibers on the outside, since failure occurs in outer layers, maximizes load bearing and impact properties. Nuryanta et al. [39] found the highest flexural strength (223.3 MPa) in the G2/A2/G2 sequence, justifying sequence significance. Outer glass fibers enhance strength with improved mechanical properties. Delamination and pull-out indicate low interfacial adhesion between fibers and matrix, limiting performance. Improved interfacial adhesion can improve composite strength further, eliminating such failure modes and improving mechanical performance.

Figure 4a illustrates load–displacement curves of the flexural behavior of different composite materials under bending stress. The curves refer to each composite combination described by the legend. This allows their mechanical performance in terms of load capacity, displacement, energy absorption, and failure mode to be compared. These curves allow for the mechanical properties of the composites and, furthermore, show how different compositions affect their structural response in the case of flexural loading. All of the peaks correspond to the maximum load that each composite will bear prior to its failure. Thus, for example, the black curve labeled [C]_8_, illustrating a bearing load of over 300 N, stands for this being the strongest composition out of the ones tested. Other configurations show their maximum load in lower values, stating that one of the most influential causes of difference in flexural strength is related to the differences in the composition of the composites themselves. The large horizontal extent of each curve corresponds to the displacement value, which is indicative of either material ductility or material brittleness. This would correspond to a brittle fracture, where for instance, after an abrupt drop in load below the peak value, as for the curve [C]_8_, the material fails suddenly with no considerable deformation; while, in contrast, a curve like the [B]_8_ shows a more gradual descent during the loss of load-carrying capability. In Figure 4a, the flexural load–displacement curve of [B]_8_ demonstrates a gradual slope indicating relatively lower stiffness due to basalt fibers’ inherent flexibility and ductile nature. Unlike composites dominated by carbon fibers, which exhibit brittle behavior and steep load increases, basalt fibers gradually bear the load, allowing higher displacements and deformation before ultimate failure. Generally, toughness is the energy that the composite absorbs under bending; it is represented by the area under each curve. Composites that normally take a higher amount of energy absorption have much higher toughness, such that they can resist fracture when high load is applied. For example, the broad curves for [B]_8_ and [B_2_C_4_B_2_] configurations indicate higher energy absorption, reflecting better toughness under bending stress. This area here is calculable through the use of numerical integration techniques, such as the trapezoidal rule, which precisely quantifies the value of energy absorbed by each composite to the point of failure. With regard to toughness, this value is important in applications where the resistance to impact is of paramount consideration.

To mathematically analyze the flexural properties, we can apply standard formulas from material mechanics. The flexural stress (*σ_f_*) in each specimen can be calculated using the formula below, Equation (1).

(1) $$\sigma_{f}=\frac{3PL}{2bd^{2}}$$

where P is the applied load, L is the support span, b is the width, and d is the thickness of the specimen. This stress value quantifies the material’s strength under bending. Similarly, the flexural strain (*ϵ_f_*) at the surface can be determined by Equation (2);

(2) $$\varepsilon_{f}=\frac{6Dd}{L^{2}}$$

where D is the midpoint deflection, representing the degree of deformation. Additionally, the flexural modulus (*E_f_*), which indicates the material’s stiffness, can be calculated from the initial linear portion of each curve using the formula below, Equation (3);

(3) $$E_{B}=\frac{L^{3}m}{4bd^{3}}$$

where m is the slope of the linear part of the curve. These properties give a quantitative comparison of the relative stiffness and strength for each composite configuration. Thus, polynomial fitting may be applied to the load–displacement data in order to approximate the specific equations for each curve, especially within the linear and peak regions. The curve can then be expressed as a polynomial function in Equation (4).

P(x) = a_n_x^n^+a_n−1_x^n−1^+⋯+a_1_x+a_0_ (4)

where P(x) is the load as a function of displacement x, and ai can be obtained using regression analysis. The ai coefficients in Equation (4) are polynomial regression constants, each indicating the contribution of a certain power of displacement to the load–displacement behavior of composites. This polynomial model can provide a mathematical representation of each composite’s flexural behavior, aiding in predictive modeling and comparative analysis. Calculations of these parameters require accurate numerical data points of each curve. Such data will allow further refinement in the polynomial models, the determination of the modulus of elasticity, and the exact energy absorption for every configuration of the composite for a final thorough understanding of their performance under flexural loading.

Figure 4b demonstrates how the flexural modulus of basalt/carbon hybrid composites is influenced significantly by the stacking sequence of the fibers. It is evident from the data presented that composites with carbon fibers positioned predominantly or entirely on the outer layers exhibit the highest flexural modulus, reaching up to 12.1 GPa. This high modulus is a result of rigidity and high stiffness inherent in carbon fibers, which have a high resistance to deformation when on external surfaces and hence can maximize their load-carrying capacity. Composites with basalt fibers on external surfaces, however, showed a much lower flexural modulus as a result of basalt fiber’s inherent ductility and comparatively lower stiffness. Basalt fibers’ increased ability to deform under bending lowers overall stiffness and modulus. Interestingly, balanced or symmetrical stackings such as [C_4_B_4_] and [B_2_C_4_B_2_] resulted in a mid-range flexural modulus, suggesting a balance between stiffness and ductility. These balanced hybrid systems maximize mechanical performance with effective stress distribution in stiff carbon layers and ductile basalt layers and hence avoid catastrophic failure and enhance structural durability. Much greater flexural modulus in the [C_2_B_4_C_2_] composite configuration is a result of stiff carbon fiber layers being placed externally, where flexural loading produces maximum tensile and compressive stresses. Carbon fibers with greater rigidity and stiffness than basalt fibers resist deformation effectively, and due to this, a sharp initial slope in the load–displacement curve is achieved and hence a much greater flexural modulus. Meanwhile, inner basalt layers provide toughness and energy absorption through delamination and fiber pull-out processes, absorbing stress but with no effect on overall stiffness. Hence, stackings have a crucial role to play in hybrid composite mechanical properties, with strategic placement of carbon fibers on the outside enhancing greatly flexural stiffness and modulus, and different configurations producing balanced mechanical properties useful for structural applications with a requirement for both rigidity and ductility.

**Figure 5:**
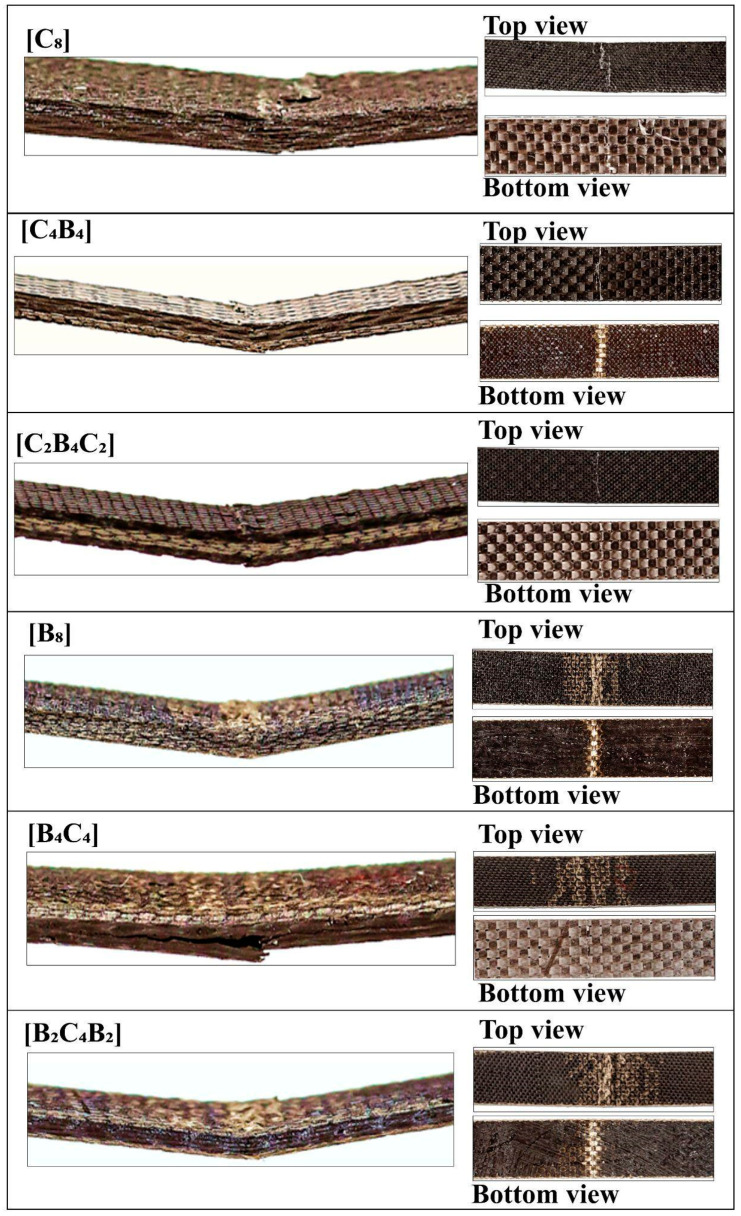
Damage images of flexural test samples.

Images of the damage types seen in hybrid basalt/carbon fiber epoxy composites during three-point bending tests are shown in Figure 5, which also illustrates particular failure mechanisms for various stacking sequences. Because of the high stiffness of carbon fiber, composites with this stacking sequence, [C]_8_, exhibit brittle fracture, with abrupt matrix breaking and little delamination. Because of the high modulus of carbon, the load–displacement curve has a steep initial slope. This is followed by a quick drop in load, which indicates catastrophic failure with little distortion. The high modulus and flexural strength numbers are consistent with this fracture behavior since carbon fibers have a limited amount of flexibility and may sustain large amounts of stress before failing suddenly. More gradual deterioration is visible in this stacking sequence [B]_8_, which shows severe matrix cracking and fiber pull-out. As evidenced by the load–displacement curve’s steady load decline, basalt’s stronger toughness and comparatively lower rigidity allow for greater energy absorption. A ductile fracture mechanism is facilitated by the higher displacement caused by basalt’s lower modulus under load. In this case, the fibers are less brittle and can absorb more energy before breaking, which is beneficial for applications that need to withstand impacts. The hybrid sequences [C_4_B_4_] and [B_4_C_4_] exhibit a variety of damage kinds. The failure modes are greatly influenced by the exterior layers, which are basalt in [B_4_C_4_] and carbon in [C_4_B_4_]. The initial load in [C_4_B_4_] is supported by the stiffness of the carbon at the outer layers, while energy dissipation is made possible by the slow matrix cracking and delamination in the interior basalt layers. On the other hand, in [B_4_C_4_], the strength of the carbon in the core layers prevents deformation, while the hardness of the basalt on the outside reduces brittle fracture, producing a balanced failure. Moderate modulus and flexural strength measurements demonstrate improved toughness and load-carrying capacity in both configurations. The stacking sequences [C_2_B_4_C_2_] and [B_2_C_4_B_2_] provide a well-balanced combination of rigidity and flexibility. With carbon on both faces, the [C_2_B_4_C_2_] structure exhibits resistance to deformation at first, but as stress grows, the basalt core gradually delaminates. This shift is highlighted by the force–displacement curve, which shows a more widespread failure with a sharp initial slope (carbon layers) and a slow fall (basalt core). Compared to carbon-dominant topologies, the [B_2_C_4_B_2_] structure, which has basalt on the outside, exhibits slow deformation that causes fiber pull-out and matrix cracking. This results in increased energy absorption but somewhat reduced flexural strength. Because carbon fibers have a low compressive strength, kink band development was noticeably more noticeable in hybrid specimens with carbon plies on the outer faces. Micro-buckling in 1C and 13C samples in uppermost layers led to a transverse crack, causing compressive failure. This was succeeded by propagation of the crack in a direction perpendicular to the direction of loading. High interlaminar shear strains led to delamination, and structural integrity was lost. With splitting in layers, surface micro-buckling initiated a multi-step process for failure, suggesting compressive limitations in arresting propagation in hybrid laminate composites [40]. The three-point bending stress–strain curve [41] is divided into three phases. First, the material is linear with a 10 GPa flexural modulus. There is 0.8–2.8% strain with irreversible deformation with loss in stiffness and visible damage on the compressive side. Tensile breakage in fibers in the third stage produces cracks and delamination in the region around the neutral axis. Carbon/aramid hybrid composites experience premature damage due to crimped structures in tows. More strain is absorbed by Specimen A (aramid weave) and a faster failure is noticed in Specimen B (carbon fibers). High displacement is noticed in Specimen C with no visible surface cracks [42].

**Figure 6:**
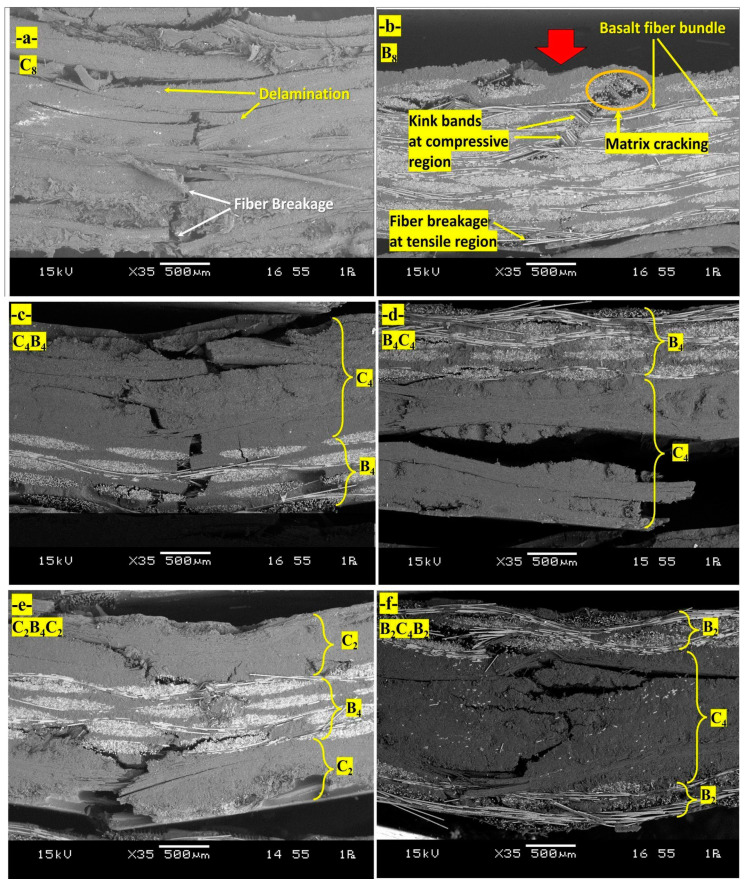
Damage mechanisms observation of hybrid composites after three point bending tests: (**a**) [C_8_], (**b**) [B_8_], (**c**) [C_4_B_4_], (**d**) [B_4_C_4_], (**e**) [C_2_B_4_C_2_], and (**f**) [B_2_C_4_B_2_]. (The red arrow shows the indenter load direction).

Figure 6a represents the mechanisms of damage in the hybrid composite with stacking sequence [C_8_] after the three-point bending tests. The fully carbon fiber configuration displays high delamination, which is a separation between layers resulting from poor interfacial bonding. The failure of the fibers in the tensile zone is related to the brittle nature of carbon fibers and reflects poor efficiency in load transfer for this type of stacking. Large areas of severe delamination show deficiencies in interfacial adhesion for this configuration. Figure 6b represents damage mechanisms in the hybrid composite with stacking sequence [B_8_], made only of basalt fiber layers. Kink bands that developed in the basalt fibers in the compressive zone reveal the low resistance of these fibers against buckling when subjected to compressive stresses. The inability of the material to bear tensile forces efficiently involved the dominant mechanism of fiber breakage in the tensile region. Visually, matrix cracking was distributed in both compressive and tensile regions, further weakening the structural integrity. Homogeneity within the stacking sequence allows for easier damage propagation due to the concentration of stress. Figure 6c represents the scheme of damage mechanisms in [C_4_B_4_] hybrid composite: every carbon and basalt fabric layer is alternating. A characteristic wide delamination has taken place almost all around the area that can be attributed to basalt fiber layers in hybrid composite with poor bonding from layer to layer, e.g., [B]_4_ and [C]_4_ regions. Simultaneously, the presence of fiber breakages related to the tensile zone evidenced relative weakness of basalt-layers in tension. In addition, the heterogeneous distribution of loads promoted by the alternation of layers is advantageous regarding the weakening matrix cracking and layer delamination processes. The mechanism of damage in the [C_2_B_4_C_2_] stacking sequence is represented in Figure 6d, showing carbon fiber layers on the outer surfaces and basalt layers in the inner core. The kink bands, developed in the basalt fibers of the compressive region, testify to their smaller stability within these inner layers under compressive stress. Higher values of tensile strength characteristic of the carbon layers suggest that much less dramatic fiber fracture occurs under tension. The matrix fracture passes along the layers with generally poor bonding between layers of carbon and basalt—the consequence of which is extreme delamination, compromising structure. Figure 6e shows mechanism of hybrid composite damage, corresponding to the stacking sequence [B_2_C_4_B_2_], with placing of basalt fiber layers outside and carbon fiber layers inside. Here, there is remarkable delamination in the interfacial regions, particularly in areas between the basalt and carbon layers due to their poor adhesion. The kink bands are prominent inside the basalt layers in the compressive region, which reflects their proneness to compressive deformation. Fiber fracture in the tensile zone is observed, while matrix cracking spreads in the layers and reduces the carrying capability of the load by the composite as a whole. Figure 6f gives the mechanisms of failure in the [B_4_C_4_] stacking with an equal number of layer alternations of basalt and carbon fiber layers; under compressive loading conditions, the kink bands arising in the compressive area will be more pronounced, which takes place for localized bending, especially in basalt fiber layers. While for the carbon layers, this has been more subdued in the tensile region, this occurs significantly in the basalt layers. The most striking factor that appeared during the flexural loading process was delamination, mostly at the basalt and carbon layer interfaces, bringing out the important aspect of interfacial bonding when it comes to the maintenance of structural integrity under flexural loading. These observations prove that the stacking sequences affect flexural damage mechanisms, extending from the development of delamination to kink band formation and breakage of fibers. The improvement of mechanical properties by improving layer arrangements and interfacial characteristics is also underlined. Table 1 presents the dominant failure mechanisms observed in basalt/carbon hybrid composites with different stacking sequences subjected to bending tests.

Tensile fracture and delamination in compression and propagation from outer to inner layers have been reported in ref. [43] as being the principal failure modes for carbon fiber composites. Kevlar composites fail in ductile buckling and glass fiber composites in buckling, delamination, and tensile fracture. CF7/GF2 and CF4/GF5 fail in CF delamination, tensile fracture in GF, and fiber pull-out. CF/KF hybrid composites fail in brittle fracture in CF and ductile failure in KF with complicated interfacial failure. Hybridization maximized fiber breakage (44%) and interfacial failure (44%), with fiber/matrix debonding (1%) and matrix cracking (9%) contributing minimally. These findings highlight hybridization to maximize composite failure behavior [44]. Ref. [45] states that composite failure is due to matrix cracking, debonding, and breakage in fibers. Acoustic emission detected damage in flexural tests with varying impact energies. Aramid laminates, being tougher, better absorbed energy and could withstand more damage than basalt laminates. Poor interfacial conditions between aramid and epoxy affected flexural behavior. Delamination and ply splitting reduced load capacity through micro-buckling and kink bands, which were visible only in compressive side glass fiber composites. Carbon fiber composites evidenced reduced ply breaking and delamination due to enhanced fiber/matrix bonding. Crushing in glass fibers and subtle but consistent damage in carbon fibers were evidenced with SEM [46].

**Figure 7:**
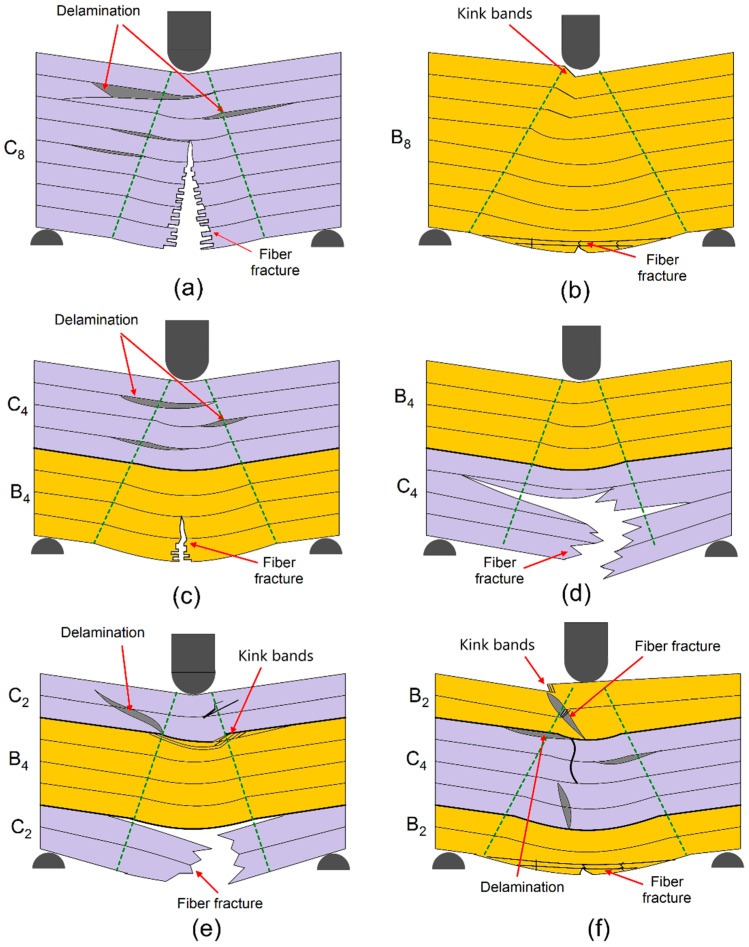
Damage mechanism schematics of hybrid composites after three point bending tests: (**a**) [C_8_], (**b**) [B_8_], (**c**) [C_4_B_4_], (**d**) [B_4_C_4_], (**e**) [C_2_B_4_C_2_], and (**f**) [B_2_C_4_B_2_]. (The green line shows the damage propagation region through the thickness).

Figure 7a shows the mechanisms of damage in a fully carbon fiber configuration [C8]. The massive delamination of the layers, resulting from poor bonding between the surfaces at the interface, accounts for much inefficiency due to an inability of proper load transfers. Fractures of the fibers, representing their brittleness, can be viewed in the tensile zone. The suddenness of this nature of response from the structural flexural load has a negligible energy dissipation capacity and falls into a category called a catastrophic mode of failure. This behavior underlines the need for improved bonding with more balanced material properties for better performance. Figure 7b shows the damage mechanism for a pure basalt fiber configuration ([B_8_]). It can be observed from this figure that the compression region is dominated by the high incidence of kink bands; indeed, this fact has been known to reflect the low buckling resistance of basalt fibers in compression. Additionally, both compression and matrix cracking were seen to appear in tension, whereas basalt fibers tend to fracture under flexural tensile stress due to limited tensile strength. In contrast, homogeneity in stacking would facilitate a rather concentration of stress in making the structure further vulnerable to the progression of such damages due to flexural action. Figure 7c shows the mechanism of damage in the hybrid composite with alternating carbon and basalt layers, [C_4_B_4_]. Delamination is widely observed, especially between basalt/carbon fiber layers, due to poor interfacial bonding. Fiber fractures in the tensile zone are associated with the weaker tensile properties of basalt layers. Despite these weaknesses, the alternating stacking sequence develops a more heterogeneous distribution of stresses that reduces matrix cracking and delays the progress of damage compared to pure basalt or carbon configurations. The mechanism of damage in the hybrid composite, with carbon fibers in the outer layers and basalt fibers in the core, [C_2_B_4_C_2_], is presented in Figure 7d. Carbon layers ensure stiffness and resistance to deformation in the tensile region. The basalt core, being less stable under compressive stresses, develops kink bands in the compressive zone. Extensive delamination was observed in this configuration at the interfaces of carbon and basalt layers, which affected structural integrity. Such a configuration can be considered optimum because it possesses a balance between rigidity and flexibility but still needs some enhancements in interfacial bonding for better performance. Figure 7e shows the mechanisms of damage in hybrid composite with basalt layers on the outer surfaces and carbon layers in the core ([B_2_C_4_B_2_]). Delamination is dominant at the interfaces between the basalt and carbon layers, which may be attributed to their mechanical property mismatch. Kink bands form in the basalt layers under compressive loading, while fiber fractures are notable in the carbon layers within the tensile region. The matrix crack propagates across the layers and reduces the effective load-carrying capability of the composite. Figure 7f illustrates the mechanisms of failure in hybrid composite [B_4_C_4_] with an equal number of basalt and carbon fiber layers. The compressive loading has caused the more pronounced development of kink bands in basalt layers because of its tendency toward localized buckling. Delamination at basalt-carbon layer interfaces was also significant, reducing further the structural integrity of the composite. Although carbon layers show better tensile resistance, the stacking sequence underlines how important interfacial bonding is in maintaining overall performance under flexural stresses.

**Figure 8:**
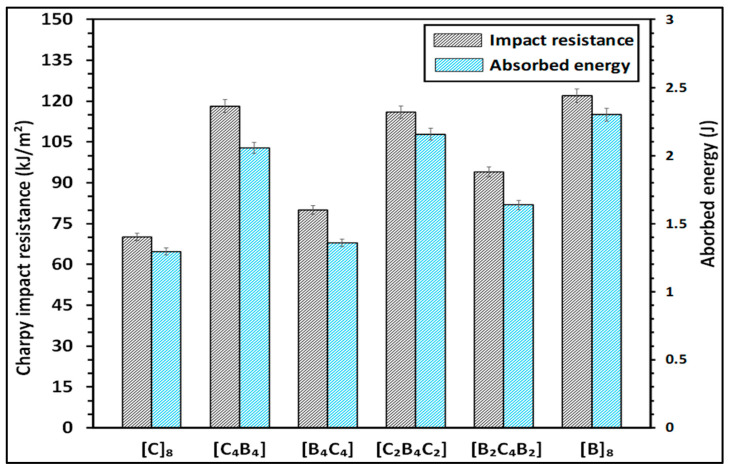
Charpy impact resistance and absorbed energy comparison of the composites.

Figure 8 compares the Charpy impact resistance and absorbed energy of the composites. Charpy impact resistance (R) is the resistance that characterizes the toughness of the material against sudden impact loads; it is expressed in kJ/m^2^. The formula for determination is Equation (5):

(5) $$R=\frac{E}{A}$$

where E is energy absorbed during fracture of the specimen in kJ, A is area, in m^2^, through which the fracture has taken place. The more the value of Charpy impact resistance, the better the performance under such impact loading, as the material is more capable of absorbing and dissipating energy with less chance for catastrophic failure.

The absorbed energy (E) in joules (kJ) quantifies the amount of energy the material applied to resist fracture during the impact test. This energy can be determined by the difference between the potential energy of the impactor before and after the test or calculated using the formula Equation (6);

(6) E=W . h

where W is the weight of pendulum (N), and h represents a difference in height of the pendulum before and after impact (m). The higher the value of absorbed energy, the tougher and more ductile the material is, since toughness involves capabilities of plastic deformation in preventing a running crack under sudden impact—this property is of vital importance in those areas which deal with energy absorption and mechanical resilience.

Figure 8 shows Charpy impact resistance and energy absorbed for the hybrid composite configurations with carbon fiber [C] and basalt fiber [B] layers in different stacking sequences. The histogram plots the left *y*-axis versus the impact resistance in kJ/m^2^ and the absorbed energy in joules on the right *y*-axis. Among the different configurations, [C_4_B_4_] and [B_2_C_4_B_2_] show the maximum impact resistance values, around 120 kJ/m^2^ and 110 kJ/m^2^, respectively. This can be understood to mean that in an alternating configuration of carbon and basalt fibers, the composite is better able to resist fracture under impact loading. Configurations of pure carbon [C_8_] and basalt [B_8_] fiber configurations present the lowest impact resistance values at approximately 75–80 kJ/m^2^. This means that interply hybrid of carbon and basalt layers—enhances the impact tolerance of the fabrics. In the same direction, the change in absorbed energy also varies, with [C_4_B_4_] and [B_2_C_4_B_2_] having absorbed the highest energy; these configurations are effective in dissipating impact energy, probably due to greater interfacial interactions and structural integrity. It has been shown that both the impact resistance and energy absorption can be significantly enhanced by the optimization of layer arrangement in composite sheets. Basalt and aramid composite laminates were tested in terms of relative energy absorption to evaluate hybrid arrangements. The energy absorption values were determined as 6.14 J and 2.80 J for the neat basalt laminate and the neat aramid laminate, respectively. In all cases, the neat basalt laminate outperformed by up to 119.29%. While aramid fibers typically possess higher energy absorption capacity compared to basalt fibers, in this case, better performance could be demonstrated due to the unidirectional structure of basalt textiles. On the other hand, the different structure may provide an explanation for the observed difference since the woven aramid textiles already have lower ultimate strength and stiffness compared to the unidirectional basalt. Ref. [28] suggests that fabric structure influences mechanical response and energy absorption in composites. Due to asymmetric laminate construction, hybrid samples underwent impact testing in two configurations: (a) Kevlar-side and (b) glass-side impact. Impact strength and absorbed energy results showed a positive hybrid effect, with Kevlar fibers enhancing impact resistance in glass fiber-reinforced composites. Hybridization with Kevlar and polyethylene fibers increased toughness and durability immensely and hence composites were more suited for use in impact applications. These findings highlight the effectiveness of fiber hybridization in enhancing the impact behavior of typical glass fiber-reinforced composites [47]. Higher energy absorption was noted in basalt composites with more layers than in glass composites. Glass composites indicated greater energy absorption with greater notch angles, and basalt composites indicated lower energy absorption. Aramid fiber samples with outer layers indicated greater energy absorption, and samples with carbon fibers indicated minimum energy absorption. All this proves that material in the outer layers is responsible for energy absorption and impact resistance in composite materials [48]. A 45° fiber orientation indicated maximum Charpy impact strength, and a 0° orientation indicated maximum Izod impact strength. A 60° orientation indicated minimum in both tests. A 0° orientation registered highest interlaminar shear strength (ILSS) with 15% and 40% improvements in 45° and 60°, respectively. These gains are a result of balanced fiber alignment in 45° Kevlar layers [49]. Basalt/epoxy laminates absorb much more energy than other variants. Basalt epoxy absorbed 34% more energy in a direction perpendicular to a flat face and 24% more in a direction perpendicular to an edge. Additional layers increased energy absorption further, showcasing basalt fiber’s remarkable impact resistance and dissipation behavior in composite applications [50].

**Figure 9:**
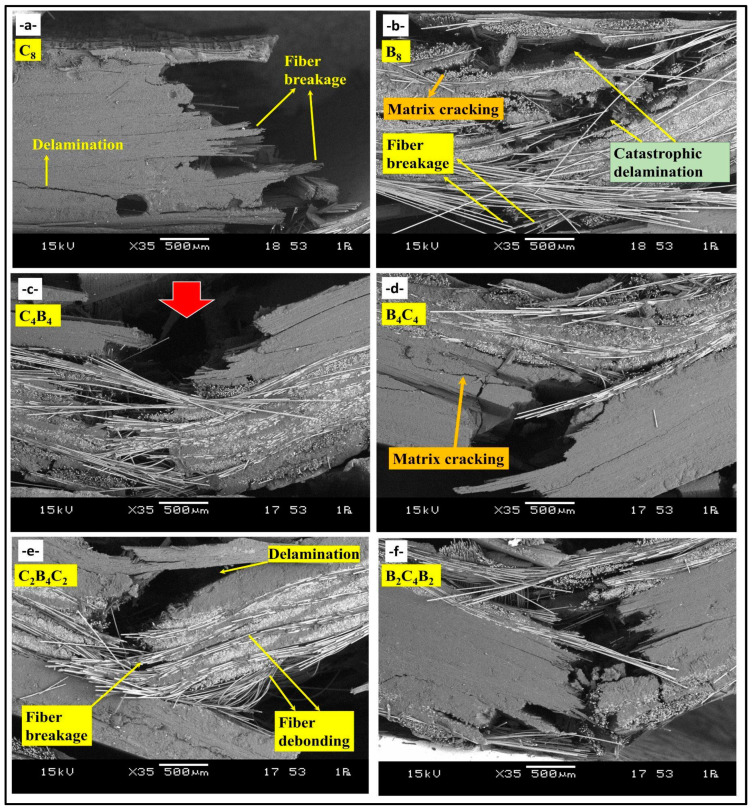
Damage mechanisms observation of hybrid composites after Charpy impact tests: (**a**) [C_8_], (**b**) [B_8_], (**c**) [C_4_B_4_], (**d**) [B_4_C_4_], (**e**) [C_2_B_4_C_2_], and (**f**) [B_2_C_4_B_2_]. (The red arrow shows the load direction of impact hamer).

Impact damage mechanisms for hybrid composite with [C_8_] stacking sequence, as highlighted in Figure 9a, are dominated by the presence of delamination between layers due to weak interfacial bonding arising from the flexural stress introduced by a Charpy impact test that is expected from its fully carbon fiber composition. This dominant mechanism here involves fiber breaks in the tensile zone. These events result due to the brittle nature of carbon fibers. The absence of other fiber types limits energy absorption and facilitates the process of catastrophic failure in the form of localized delamination and extensive fiber fracture. Figure 9b illustrates damages in the basalt fiber only stacking sequence of [B_8_]. The damage, as observed from this figure, consists of quite a lot of matrix cracks and fiber breakage in tension and compression parts of this specimen. Large catastrophic delamination can be observed, which grows along the fiber–matrix interface because of the weaker bonding between basalt fibers and resin. In addition, the kink bands in the compressive region demonstrate the basalt fibers that deformed under the impact loading have buckled, which points out a very low resistance to compression, much smaller compared to carbon fibers. Figure 9c illustrates the failure mechanisms of the hybrid composite [C_4_B_4_], whose carbon and basalt fibers were laid up in a layer-by-layer alternate pattern. A red arrow indicates that heavy delamination was even further concentrated by the stacking pattern, [C_4_B_4_], since it provides a stress concentration between pure carbon and basalt plies. The pull-out behavior of the fibers indicates loss of sufficient adhesion between the fibers and matrix. The impact in the Charpy creates a combination of matrix cracking, fiber fracture, and delamination, underlining the challenges in balancing the mechanical performance of such a hybrid structure. Figure 9d illustrates the stacking sequence [B_4_C_4_]; layers of basalt and carbon fibers are stacked in the reverse order of what was indicated in Figure 9c. It is observed from this image that matrix cracking occurred near the fiber interfaces. The delamination was found spreading between layers. The stacking sequence presents similar effects of stress concentration at the interface but evidence of better load sharing compared to a fully basalt or carbon structure. However, due to the matrix’s limited ability to effectively absorb impact energy, fiber breaks and crack propagations remain prominent. Figure 9e illustrates impact damage mechanisms in [C_2_B_4_C_2_] stacking sequence with the presence of basalt fiber layers sandwiched between outer carbon layers. The delamination is pronounced at interfaces between carbon and basalt fiber layers, indicating weak bonding of this arrangement. There is distinct fiber breakage in both the tensile and compressive zones, while there was significant fiber pull-out present in the basalt layers. The Charpy impact test emphasizes the mismatch in properties between the carbon and the basalt layers because extensive matrix cracking and consequent loss of structural integrity are encountered. Figure 9f illustrates the damage in the stacking sequence [B_2_C_4_B_2_], with carbon fiber layers internally and basalt fibers on the outside. In all these mechanisms, matrix cracking and debonding of fibers near basalt fiber layers dominate. Fiber fracture seems to occur only in carbon fiber areas whereas basalt fibers show pull-out due to poor bonding with the matrix. This results in significant delamination, driven by the differences in stiffness and energy absorption capabilities of carbon and basalt fibers. Such a stacking sequence illustrates the limitations of hybrid configurations under impact loading conditions since the stress redistribution through the layers is not uniform.

Table 2 presents the dominant failure mechanisms observed in basalt/carbon hybrid composites with different stacking sequences subjected to impact tests.

**Figure 10:**
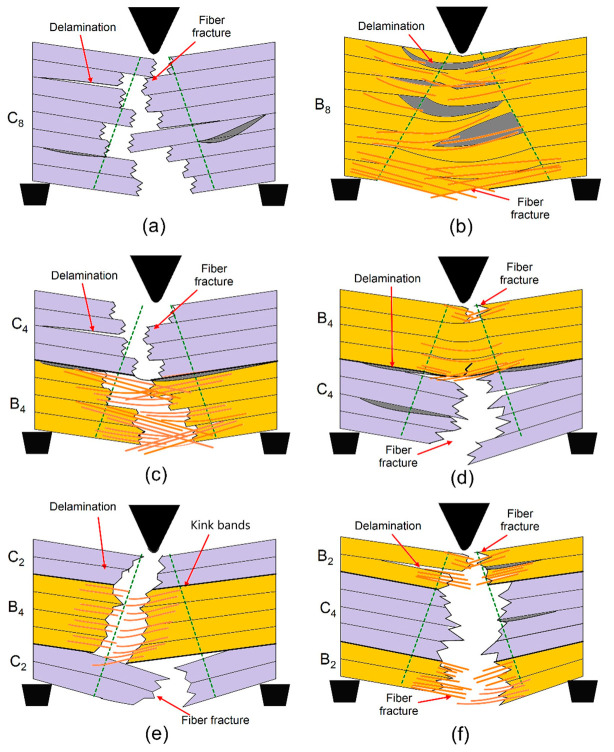
Damage mechanism schematics of hybrid composites after Charpy impact tests (**a**) [C_8_], (**b**) [B_8_], (**c**) [C_4_B_4_], (**d**) [B_4_C_4_], (**e**) [C_2_B_4_C_2_], (**f**) [B_2_C_4_B_2_]. (The green line shows the damage propagation region through the thickness).

Figure 10a presents the mechanisms of damage in fully carbon fiber ([C_8_]) configuration under impact loading. The presence of weak interfacial bonding between layers initiates the major mechanisms of delamination. With brittle carbon fibers, there is a higher number of total fiber fractures in a tensile zone that suppresses the energy dissipation characteristics under impact for this class of material. The full absence of ductile fiber further promotes a stress concentration point promoting local failures and subsequent catastrophic damage growth. Figure 10b shows the mechanisms involved in the failure of the fully basalt fiber configuration ([B_8_]). Large matrix cracking is visible, both in tensile and compressive zones. Fiber breakage is dominant in the tensile zone, while in the compressive zone, kink bands reveal that basalt fibers tend to buckle under dynamic loadings. The delamination along the interface between fibers and matrix provides further weakness in the structure and results in lower energy absorption capability. Figure 10c displays the damage mechanisms occurring in the hybrid composite with stacked layers of the alternating carbon and basalt, [C_4_B_4_]. The stacked sequence creates the strain localization at the interfaces between the carbon and basalt layers and thus significant delamination. Fiber pull-out and matrix cracking has been seen predominantly because of poor adhesion between the fibers and matrices. With the alternate layers of improvement, the hybrid structure is unable to keep the balance of stiffness and energy absorption qualities under impact loading conditions. Figure 10d illustrates the damage mechanisms in the reverse stacking sequence [B_4_C_4_], with the basalt fibers on the outside and carbon layers inside. In the impact loading, matrix cracks near the fiber interfaces and heavy delamination takes place in this sequence. Although the stress redistribution in this case is somewhat improved when compared to the fully basalt or fully carbon configuration, still quite a lot of fiber fractures and crack propagation is developed since the matrix can absorb a limited amount of energy. Figure 10e shows the mechanisms of hybrid composite damage with basalt fibers in between outer layers of carbon ([C_2_B_4_C_2_]). In this case, the presence of carbon on the outside gave rigidity and resistance in the initial phases of deformation. However, there is very pronounced delamination at the interface between the basalt and carbon layers. A few fiber fractures were observed in tension and compression, with large pull-out of the basalt fibers. The large difference in properties between these two types of fibers results in severe matrix cracking with loss in structural integrity. The fractal mechanism is represented in the hybrid composite where the carbon fibers are sandwiched by the basalt fiber outer layers ([B_2_C_4_B_2_]), as observed from Figure 10f. The external fibers, in the present case basalt fibers, consume a portion of the impact energy through ductile deformation; still, fiber pull-out and matrix cracking are two main damage modes under tension. Furthermore, massive delamination events have taken place at the interface. Such cases would be attributed to the big gaps in terms of stiffness and/or dissipation potential that may occur at the interface, with the coexistence of two fibers: one carbon and another basalt. The above stacking sequence illustrates the difficulty of uniform stress redistribution in impact conditions. These observations confirm the fact that the dynamic impact load improves the mechanisms of damage in the flexure tests. The sequence of the different layers has an important place in the failure modes; indeed, hybrid configurations showed mixed modes involving delamination, fiber fracture, and kink band formation. Energy absorption can only be improved together with limiting the damage propagation under the impact loading conditions in the hybrids, and these depend upon two main variables of optimization: the layer arrangement and the fiber–matrix adhesion.

**Figure 11:**
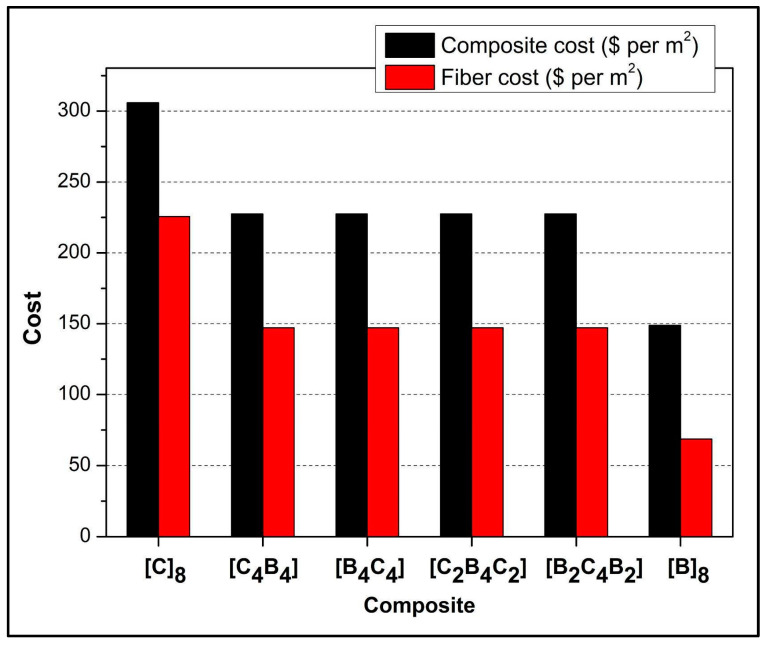
Cost comparison of manufactured composite samples.

Figure 11 demonstrates cost analysis of composites showing the economic and environmental advantages of using basalt fiber as an alternative to carbon fiber. The chart shows that the cost of carbon fiber is relatively high; for example, the pure carbon fiber composite [C]_8_ has a total cost of approximately $300/m^2^, with a significant portion attributed to fiber cost. In contrast, the pure basalt fiber composite [B]_8_ has the lowest total cost at around $150/m^2^. As the proportion of basalt increases, there is a noticeable reduction in composite costs; for instance, the composite with equal parts carbon and basalt [C_4_B_4_] is less expensive than the pure carbon fiber composite. This reduction is primarily due to the relatively lower unit price of basalt fiber compared to carbon fiber. The cost of epoxy resin is nearly the same for all the composites; therefore, the differences in cost will predominantly involve the type of fiber applied. From an environmental perspective, basalt fiber is renewable, abundant, and takes less energy to produce compared to carbon fiber; this leads to lesser greenhouse gas emission. These qualities, therefore, make basalt a greener and cheaper alternative, mainly for cost-sensitive and green-conscious industries like automotive and construction. The prices of the available materials are as follows: basalt, $8.58/m^2^; carbon fiber, $28.2/m^2^; epoxy resin, $34.9/kg; and hardener, $50.92/kg [51,52].

**Figure 12:**
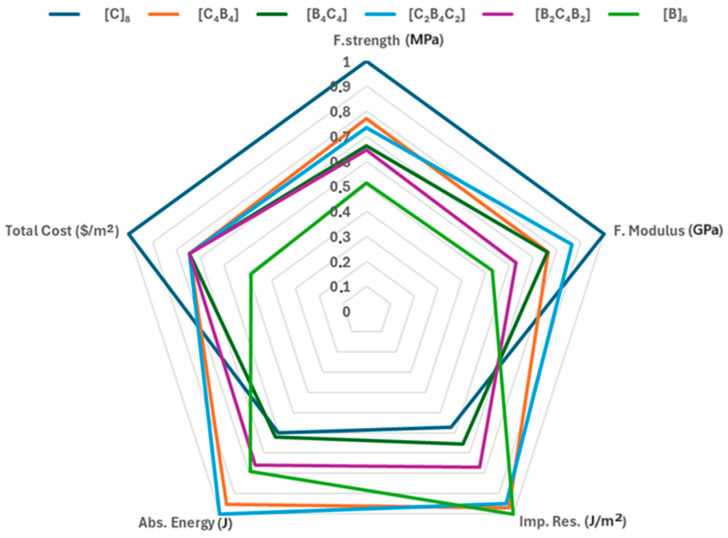
Total cost, flexural strength, flexural modulus, impact resistance and absorbed energy comparison of manufactured composite samples.

Figure 12 compares in detail all the composite samples prepared for some key parameters like total cost, flexural strength and modulus, impact resistance, and absorbed energy. Every line-color-coded according to the legend-specifies a certain layer configuration and reinforcement type: [C]_8_, [C_4_B_4_], [B_4_C_4_], [C_2_B_4_C_2_], [B_2_C_4_B_2_], and [B]_8_. A significant difference is observed among the configurations. The flexural strength and modulus of the composite [C]_8_, represented by the dark blue line, is the highest among the combinations considered. That would signify that it has the highest resistance to bending forces and is suitable for load-carrying applications. Unfortunately, there is also a correspondingly relatively high cost for this configuration, placing it as a premium choice. On the other extreme, the [B]_8_ composite is colored green and displays the lowest cost, its economically favorable value being an advantage. The result of such a combination is a deficiency in flexural strength and modulus, which could mean it is not as structurally strong as some other combinations. Further explaining their mechanical properties, the absorbed energy and impact resistance in this configuration is at mid-level-balance cost with satisfactory resilience to impacts. Configuration [C_4_B_4_] and [B_4_C_4_], represented by an orange and green line, respectively, show similar magnitudes of flexural modulus and impact resistance. These two geometries represent a trade-off between cost and strength, making them intermediate configurations. However, the [C_4_B_4_] configuration shows slightly better performance in absorbed energy, which may imply that it could provide better energy dissipation in case of an impact; this will result in enhanced toughness, as often sought in engineering applications. The colors light blue and purple are given to the geometries [C_2_B_4_C_2_] and [B_2_C_4_B_2_], respectively, reporting a more balanced behavior for the three parameters being considered. These configurations represent a balance in moderate levels of flexural strength, modulus, impact resistance, and absorbed energy that provides versatile choices balancing mechanical performances with cost consideration. It finds applicability for those situations where the application requires a blend of properties without extreme peaks in any one characteristic. This comparative analysis brings out the trade-off relationship between cost and mechanical properties in manufactured composite samples. The structure [C]_8_ is advised for applications needing ultimate tensile strength, whereas [B]_8_ is more viable in cost-sensitive projects. Configurations such as [C_4_B_4_], [B_4_C_4_], [C_2_B_4_C_2_], and [B_2_C_4_B_2_] are balanced structures suitable for most applications that need a moderate mix of the mechanical and economic attributes. Hybrid laminates were tested for specific strength, stiffness, and cost-effectiveness. CFRP laminates outperformed hybrids in strength and stiffness but are more expensive. The comparison highlights that hybrid laminates are more cost-effective, though CFRP is preferable for applications requiring higher strength and stiffness despite the higher cost. The findings confirm cost and performance requirement-based laminate selection [53]. Plain and hybrid composite cost-performance balance was determined with flexural strength, modulus, and impact strength. Plain carbon fiber composites deliver best performance, and plain glass fiber composites are cost-effective. The hybrid stacking sequence [CGKCKGC] shows better cost efficiency than [CKGCGKC], making it suitable for applications where high strength/cost ratio is crucial, such as bumpers and car bodywork [54]. Glass/carbon hybrid composites showed higher material efficiency in both strength-to-cost and modulus-to-cost ratios. These cost-effective benefits make them suitable for applications where cost and performance are important. The combination of FEM and experimental techniques provided accurate performance predictions, promoting the use of hybrid composites in structural design applications [55]. Glass and/or basalt fibers enhance the mechanical performance of flax-reinforced epoxy composites. This hybrid product has lower cost and lighter weight with comparable strength. Flax-based hybrid composites are most suited to be used in mid-level loads where cost and performance are crucial and can be a good substitute for pure glass or basalt fiber composites for modest levels of strength without added cost or weight [56].

## 4. Conclusions

In this work, six different stacking sequences of basalt/carbon epoxy hybrid composite mechanical properties were evaluated under flexural and impact loading conditions. The results of this work indicated significant improvement in the mechanical properties upon hybridization compared to that of single-fiber configuration. Composites with outside layers of carbon fiber, like [C_2_B_4_C_2_], offered the maximum flexural strength and modulus of 357 MPa and 12.1 GPa, respectively, due to the rigidity and stiffness provided by carbon fibers. By contrast, hybrid structures with basalt fibers as outer layers, such as in [B_2_C_4_B_2_], indicated much higher energy absorption corresponding to impact resistance up to 110 kJ/m^2^, thus underlining that basalt fibers have some ductile nature. Whereas in balanced hybrid configurations both [C_4_B_4_] and [B_2_C_4_B_2_] present an optimal blend of stiffness and toughness within the framework of strength vs. energy absorption. Results by SEM analysis after failure indicated that the delamination and pull-out of fibers were the prevalent damage mechanisms; its impact is greatly influenced by stacking sequence with regard to distribution of stresses. Carbon layers improve resistance to compressive as well as tensile failures, while basalt layers contribute to an increased energy dissipation under impacting situations. The addition of basalt fibers reduces the overall price by up to 40%, hence making the basalt-based hybrids developed herein cost-effective while retaining properties. The improvements in the quality of basalt-carbon interfacial bonding are expected to alleviate delamination and poor mechanical performance in the future. Further, studies dealing with nano-reinforcement or new resin additions will lead to better fiber–matrix adhesion and further durable performance. Numerical modeling and optimization of hybrid stacking sequences will further refine the cost-performance balance for particular applications of the aerospace and automotive industry. The environmental benefits of the basalt fibers also have applications in studies on sustainability and life-cycle analysis for the hybrid composites mentioned.

## Figures and Tables

**Table 1:** The dominant failure mechanisms of bending loading.

Specimen	Dominant Failure Mechanism in Bending
[C_8_]	Brittle fracture, abrupt matrix cracking, limited delamination
[B_8_]	Severe matrix cracking, fiber pull-out, ductile failure
[C_4_B_4_]	Wide delamination, fiber breakage in basalt layers, matrix cracking
[B_4_C_4_]	Delamination between layers, kink bands in basalt layers, balanced fiber breakage
[C_2_B_4_C_2_]	Kink bands in basalt core, delamination at carbon-basalt interfaces
[B_2_C_4_B_2_]	Fiber pull-out, matrix cracking, significant delamination at basalt-carbon interfaces

**Table 2:** The dominant failure mechanisms of impact loading.

Specimen	Dominant Failure Mechanism in Impact Loading
[C_8_]	Fiber fracture, extensive delamination, brittle fracture
[B_8_]	Matrix cracking, extensive fiber breakage, kink bands, and catastrophic delamination
[C_4_B_4_]	Fiber pull-out, matrix cracking, significant delamination concentrated at basalt-carbon interfaces
[B_4_C_4_]	Delamination at layer interfaces, matrix cracking, moderate fiber breakage
[C_2_B_4_C_2_]	Extensive delamination at basalt-carbon interfaces, pronounced fiber breakage, basalt fiber pull-out
[B_2_C_4_B_2_]	Delamination at interfaces, fiber pull-out, significant matrix cracking, and kink bands

## Data Availability

The datasets presented in this article are not readily available because the data are part of an ongoing study. Requests to access the datasets should be directed to Corresponding Author.

## Abbreviations

The following abbreviations are used in this manuscript:

FRPMC	Fiber-reinforced polymer matrix composites
CFRP	Carbon fiber reinforced polymer
